# Reliability of neuronal information conveyed by unreliable neuristor-based leaky integrate-and-fire neurons: a model study

**DOI:** 10.1038/srep09776

**Published:** 2015-05-13

**Authors:** Hyungkwang Lim, Vladimir Kornijcuk, Jun Yeong Seok, Seong Keun Kim, Inho Kim, Cheol Seong Hwang, Doo Seok Jeong

**Affiliations:** 1 Electronic Materials Research Center, Korea Institute of Science and Technology, 136-791 Seoul, Republic of Korea; 2 Department of Materials Science and Engineering and Inter-university Semiconductor Research Center, Seoul National University, 151-744 Seoul, Republic of Korea; 3 Department of Materials Science and Engineering, Seoul National University of Science and Technology, 139-743 Seoul, Republic of Korea

## Abstract

We conducted simulations on the neuronal behavior of neuristor-based leaky integrate-and-fire (NLIF) neurons. The phase-plane analysis on the NLIF neuron highlights its spiking dynamics – determined by two nullclines conditional on the variables on the plane. Particular emphasis was placed on the operational noise arising from the variability of the threshold switching behavior in the neuron on each switching event. As a consequence, we found that the NLIF neuron exhibits a Poisson-like noise in spiking, delimiting the reliability of the information conveyed by individual NLIF neurons. To highlight neuronal information coding at a higher level, a population of noisy NLIF neurons was analyzed in regard to probability of successful information decoding given the Poisson-like noise of each neuron. The result demonstrates highly probable success in decoding in spite of large variability – due to the variability of the threshold switching behavior – of individual neurons.

The human brain—three pounds of matter between our ears—has not yet been understood completely because of its complexity. For many decades, researchers have focused on understanding the principles and detailed actions of the human brain and, in general, the mammalian brain^1^,^2^,^3^,^4^. The unique functionalities of the mammalian brain, such as parallel information processing, low power consumption, and learning capacity, make it fascinating. These unique functionalities are of great interest to not only neuroscientists but also physicists and electrical/materials engineers. There have been many attempts to realize “artificial brains” either by hardware- or software-based techniques^5^,^6^,^7^,^8^,^9^,^10^. In particular, the latter is often termed as *in silico* neural network^11^. Here, the term ‘artificial brain’ denotes an electronic system that mimics some limited neural functionalities. In general, such efforts are often referred to as neuromorphic engineering, a phrase coined by Carver Mead^5^.

The basic elements in a mammalian brain—a complex neural network—are neurons and synapses; synapses define the connectivity between neighboring neurons, and function as local memories^12^. Synapses and neurons are the basic elements in artificial neural networks (ANNs) as well. Note that in this study ANN denotes both hardware- and software-based networks. Neurons are of significant importance as they generate action potentials (also known as spikes), and work as information units in neural networks^2^. Various types of artificial neuron models can be employed in ANNs, such as leaky integrate-and-fire (LIF) neuron^1^,^2^,^3^, Hodgkin-Huxley neuron^13^, and Izhikevich neuron models^14^,^15^. Among these, the LIF neuron is the simplest model and can be easily implemented in ANNs^1^,^2^,^3^.

To date, a great deal of efforts have been made to realize the different types of artificial hardware neurons, including the LIF neuron, using conventional complementary metal-oxide-semiconductor (CMOS) technologies^16^,^17^. This CMOS-based approach has been the mainstream approach of neuromorphic (hardware) engineering. A recent emerging research trend in neuromorphic engineering is the increasing adoption of alternative approaches to realize artificial neurons and synapses. These emerging approaches differ from the mainstream approach in that neural functionalities are implemented by introducing functional materials-based elements that could partly replace CMOS-based elements in the former^12^. One of the advantages of these new approaches is that it may enable the circuitry of ANNs to be substantially simplified by using a less number of CMOS elements than the conventional approach. An example of such approaches is a recent breakthrough by Pickett *et al*., who achieved the LIF neuron by using two pairs of a Mott insulator and a capacitor^18^. Basically, the neuristor concept introduced by Crane^19^ in 1962 was employed in the LIF neuron model; hence this LIF neuron is termed as neuristor-based LIF (NLIF) neuron in this study so as to differentiate it from the standard LIF neuron.

Mott insulators are known to undergo temperature-driven insulator-to-metal transitions, so that the conductivity abruptly increases when the lattice temperature exceeds the transition temperature^20^. This transition is reversible, that is, the initial conductivity is recovered when the lattice temperature again falls below the threshold for the reverse transition, i.e., metal-to-insulator transition. As the lattice temperature change is due to Joule heating, the current–voltage (*I*–*V*) behavior of the Mott insulator is estimated to be volatile, i.e., threshold switching^18^,^21^,^22^. Threshold switching plays a key role in the functioning of the NLIF neuron^18^,^23^. Other than the Mott insulator, amorphous higher chalcogenides^24^,^25^,^26^,^27^, Si n^+^/p/n^+^ junctions^28^, and particular transition metal oxides such as NbO_x_^23^,^29^ are also known to exhibit volatile threshold switching.

Regardless of the type of threshold switch (TS) in the NLIF neuron, the variability of threshold switching behavior cannot be completely avoided^24^,^26^. It is, therefore, required to determine the effect of this variability on the neuronal behavior of the NLIF neuron that often leads to neuronal noises. The quantitative understanding of the noise is of significant importance when employing the NLIF neuron in both *in silico* and hardware-based ANNs because some noise properties are acceptable in ANNs insomuch as the noise does not cause serious errors during information processing^30^.

In this study, we conducted simulation on the NLIF neuron in order to identify its neuronal behavior – its noise characteristics and information representation in the presence of noise. Indeed, the employed analysis methods are widely used in characterization of other neuron models^1^,^3^,^31^,^32^, so that one can readily compare the characteristics of the NLIF neuron model with those of other models.

We first attempted to find optimized operational windows for the variables in the NLIF neuron model. It was indeed not an easy task to find the windows owing to the many variables involved simultaneously. We suggest a method to find successful spiking conditions by conducting static and dynamic calculations on the NLIF neuron circuit. The acquired windows should be narrowed down by taking into account the optimal selectivity of individual NLIF neurons for stimulation. The neuronal selectivity is one of the essential functions of neurons, given that they work as information encoders, in particular, in the presence of noise. Next, we allowed the variability of the TS in an individual NLIF neuron under optimal firing conditions. Such variability is most likely seen every switching cycle^24^,^26^—switching-event-driven variability. Threshold switching events repeatedly occur throughout the external stimulation duration, rendering the variability to feature a noise in the neuron’s response. By analyzing the noise property, the relationship between the distribution and the consequent noise is understood and compared with the noise present in biological neurons. A question arising from the analysis on individual NLIF neurons is “Can conveying information, such as encoding and decoding, be achieved in a reliable manner by a population of these individual NLIF neurons?” This question is related to neuronal behavior at a higher dimension, i.e., the group, rather than at the individual neuronal level. The Bayesian decoder was employed so as to examine the reliability of the information conveyed by a population of NLIF neurons. As a result, the reliability was evaluated by means of “uncertainty.”

## Results

### Optimal firing conditions of individual NLIF neurons

**Figure 1:**
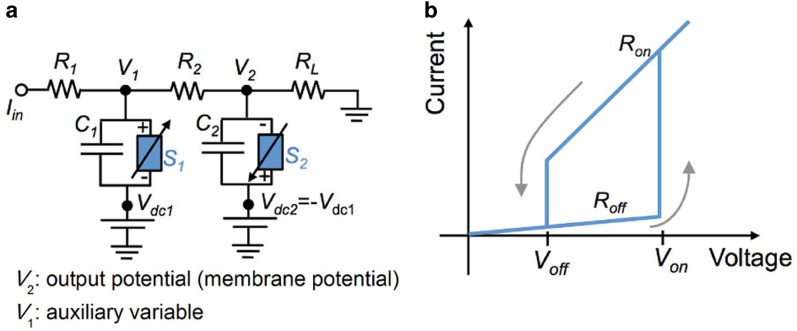
Circuitry of NLIF neuron and threshold switching behavior. (**a**) Circuitry of the NLIF neuron. (**b**) A schematic of *I−V* behavior of a TS.

In a single NLIF neuron circuit, standard circuit elements such as resistors (*R*_*1*_, *R*_*2*_, and *R*_*L*_), capacitors (*C*_*1*_ and *C*_*2*_), and TSs (*S*_*1*_ and *S*_*2*_) are in use, as shown in Fig. 1a. ^18^ When it comes to a network of NLIF neurons, *V*_*2*_ in Fig. 1a is relayed to a neighboring neuron through a synapse, so that *V*_*2*_ works as the output voltage, corresponding to membrane potential. To generate a positive spike *V*_2_, *V*_dc2_ and *V*_dc1_ need to be positive and negative, respectively. For simplicity, it is assumed that *V*_dc2_ *=* *−V*_dc1_ *=* *V*_d_. The two dc voltage sources (*V*_*dc1*_ and *V*_*dc2*_) effectively supply power – enabling active operation – and determine the spiking dynamics including the spike’s height and the undershoot level following the spike. The spiking dynamics will be explained in detail later. The key component in the NLIF neuron is the TS that performs monostable switching. The monostability of a TS can be understood from the schematic of current–voltage (*I*–*V*) hysteresis of the TS illustrated in Fig. 1b. The behavior of the TS is described by four parameters: *R*_*on*_, *R*_*off*_, *V*_*on*_, and *V*_*off*_, which denote the on- and off-state resistances and threshold voltages for off-to-on and on-to-off switching, respectively. The assumption of linear *I*–*V* behaviors in both the states allows constant *R*_*on*_ and *R*_*off*_ in a given operational voltage range. For simplicity, switches S_1_ and S_2_ are assumed to be identical.

The NLIF neuron fires spikes only when switch S_2_ flickers at a given input current (*I*_*in*_). Note that the term “flicker” means completing a threshold switching cycle, for instance, that along the arrows in Fig. 1b. To meet this requirement, the five standard circuit components (*R*_*1*_, *R*_*2*_, *R*_*L*_, *C*_*1*_, and *C*_*2*_), the four operational parameters of the TS (*R*_*on*_, *R*_*off*_, *V*_*on*_, and *V*_*off*_), and the dc voltage (*V*_*d*_), i.e., ten variables in total, should be optimized. Owing to the difficulty in optimizing such a large number of variables, it is required to rule out several variables, in particular the operational parameters of the TS, that are most likely estimated from available experimental data^24^,^26^,^27^,^29^. In this calculation, *R*_*off*_ and *V*_*on*_ were set to 1 Mohm and 1 V, respectively, so that only two variables (*R*_*on*_ and *V*_*off*_) of the TS remain. They were converted to the following normalized variables: *R*_*off*_*/R*_*on*_ and *V*_*off*_*/V*_*on*_. These threshold switching parameters are summarized in Table 1. Note that these ratios are often employed in characterizing resistive switching devices. A further reduction in the number of variables was made by setting *R*_*d*_ and *V*_*d*_ to 1 Gohm and 0.9 V, respectively. *R*_L_ works as a voltage divider in this single neuron; it is desired to be large. *V*_*d*_ needs to be close to, but smaller than, *V*_*on*_ so as to turn on switch *S*_*2*_ with a small input current *I*_*in*_; 90 percent of *V*_*on*_, i.e., 0.9 V, was taken as *V*_*d*_.

**Figure 2:**
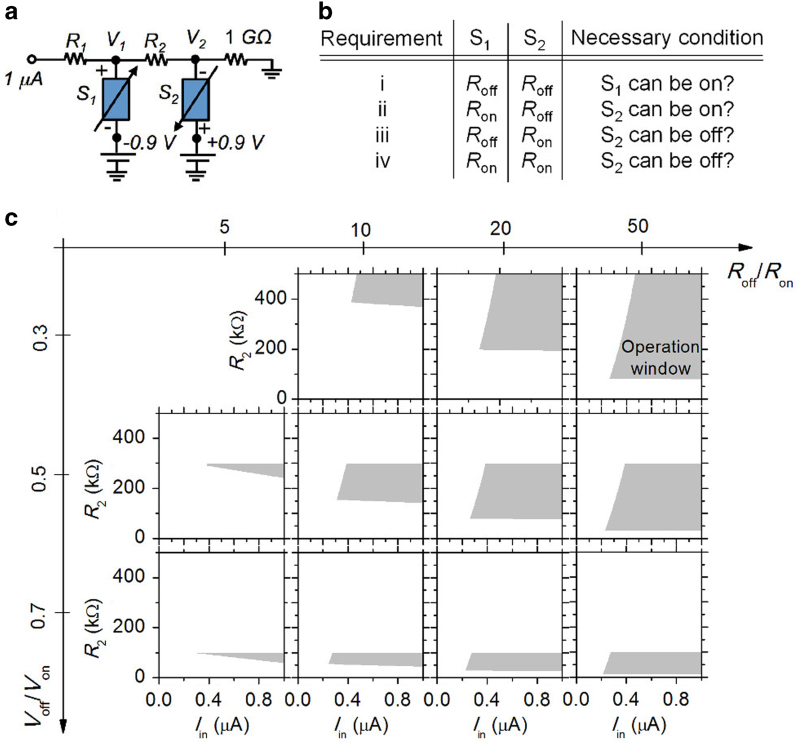
Operational windows of series resistance. (**a**) Circuitry of the NLIF neuron for static calculations. *R*_*L*_ and *V*_*d*_ are set as 1 Gohm and 0.9 V, respectively. (**b**) A table of requirements for successful spiking. (**c**) Acquired operational windows (grey zones) of *R*_2_ and *I*_in_ for successful spiking at given ratios of *R*_*off*_*/R*_*on*_ and *V*_*off*_*/V*_*on*_.

To arrive at a condition of flickering switch S_2_ at a given *I*_*in*_, time-independent calculations were performed with capacitors *C*_*1*_ and *C*_*2*_ ruled out (see Fig. 2a). The calculations provided *I*_*in*_ and *R*_*2*_ windows for spiking at given *R*_*off*_*/R*_*on*_ and *V*_*off*_*/V*_*on*_ values. The condition drawn from these static calculations is a “prerequisite” for successful spiking in the time domain. This is because the capacitors only determine the rate of voltage redistribution in the NLIF neuron upon switching of *S*_*1*_ and *S*_*2*_, and the voltages across the two switches will eventually reach *V*_*on*_. Meeting the four requirements, shown in Fig. 2b and described below, allows *S*_*2*_ to flicker. Note that on-switching of switch *S*_*1*_ is a necessary condition for that of switch *S*_*2*_, but off-switching of switch *S*_*1*_ is unnecessary for that of switch *S*_*2*_. **Requirement i**: setting *R*_*off*_ for both switches in the circuit results in a voltage across switch *S*_*1*_ (*|V*_*1*_*+V*_*d*_*|*) that is larger than *V*_*on*_, leading to the off-to-on switching of switch *S*_*1*_, given the aforementioned necessary condition for on-switching of switch *S*_*2*_. **Requirement ii**: setting *R*_*on*_ and *R*_*off*_ for switches *S*_*1*_ and *S*_*2*_, respectively, results in a voltage across switch *S*_*2*_ (*|V*_*2*_*–V*_*d*_*|*) that is larger than *V*_*on*_, leading to off-to-on switching of switch *S*_*2*_. **Requirement iii**: setting *R*_*off*_ and *R*_*on*_ for switches *S*_*1*_ and *S*_*2*_, respectively, allows on-to-off switching of switch *S*_*2*_ by decreasing *|V*_*2*_*–V*_*d*_*|* below *V*_*off*_. **Requirement iv**: setting *R*_*on*_ for both switches allows on-to-off switching of switch *S*_*2*_ regardless of on- or off-switching of switch *S*_*1*_, given that off-switching of switch *S*_*1*_ is not a necessary condition for that of switch *S*_*2*_. Satisfying these requirements, *R*_*2*_ windows with respect to input current *I*_*in*_ and combinations of *R*_*off*_* /R*_*on*_ (5, 10, 20, and 50) and *V*_*off*_* /V*_*on*_ (0.3, 0.5, and 0.7) values were obtained as indicated using the grey zones in Fig. 2c. The white zones correspond to the failure of spiking. Insomuch as a current rather than a voltage is applied, *R*_*1*_ and *R*_*2*_ are independent variables as can be seen in Fig. 2c. It is noticed that the higher *R*_*off*_* /R*_*on*_ and the lower *V*_*off*_* /V*_*on*_ ratio are, the wider *R*_*2*_ window is. For the following calculations, we chose moderate parameters of the TS (*R*_*off*_*/R*_*on*_ *=* 20 and *V*_*off*_*/V*_*on*_ *=* 0.5) and *R*_1_ and *R*_2_ of 100 kohm.

As mentioned earlier, the windows drawn from the static calculations serve as necessary, rather than sufficient, conditions for successful firing of the NLIF neuron in a time domain; therefore, capacitors *C*_*1*_ and *C*_*2*_ need be optimized as well. The only concern in spiking in due course would be the sequential on-switching events of switches *S*_*1*_ and *S*_*2*_ when both are in the off-state, i.e., aforementioned Requirements i and ii are met in consecutive order. The major role of capacitors *C*_*1*_ and *C*_*2*_ in spiking is time-dependent redistribution of *V*_*1*_ and *V*_*2*_ upon switching of *S*_*1*_ and *S*_*2*_. The capacitors determine the rate of the redistribution. That is, the higher the capacitance, the lower the rate. To satisfy the above-mentioned requirements, the time required for the evolution of *V*_*2*_—eventually leading to *|V*_*2*_*–V*_*d*_*|*≥*V*_*on*_, i.e., on-switching of switch *S*_*2*_ upon the on-switching of switch *S*_*1*_—should be sufficiently short to hinder the off-switching of switch *S*_*1*_ in the meantime. Otherwise, an increase in *|V*_*2*_*–V*_*d*_*|* in due course, owing to the on-switching of switch *S*_*1*_, would be abruptly diminished before *V*_*on*_ is reached. Note that it was assumed that the switching times of *S*_*1*_ and *S*_*2*_ are sufficiently short to have a negligible impact on the time-dependent voltage redistribution. In addition, regarding the off-switching of switch *S*_*2*_, the static calculations basically assume the instability of the on-state of switch *S*_*2*_ (see Requirements iii and iv), and thus off-switching occurs regardless of capacitances of *C*_*1*_ and *C*_*2*_.

**Figure 3:**
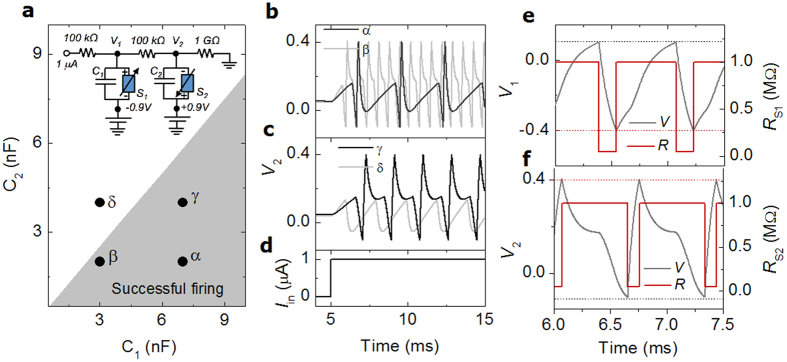
Operational windows of capacitance. (**a**) Window of *C*_*1*_ and *C*_*2*_ (grey zone) for successful spiking in due course, evaluated by time-dependent calculation. The inset shows the NLIF neuron circuit with parameters used in this calculation. Four combinations of *C*_*1*_ and *C*_*2*_, α (7 nF, 2 nF), β (3 nF, 2 nF), γ (3 nF, 4 nF), and δ (3 nF, 4 nF), are sampled and voltage-time behaviors of NLIF neurons with the capacitance combinations are plotted in (**b**,**c**). Input current *I*_*in*_ is shown in (**d**). For case β, the evolution of *V*_1_, *V*_2_, *R*_S1_, and *R*_S2_ is zoomed in in (**e**,**f**) to identify the self-consistent relation between them. The black and the red dashed line denote thresholds for on- and off-switching, respectively.

Given the above-mentioned requirements, a capacitance window for spiking at an input current of 1 μA is obtained as shown in Fig. 3a. The input current profile with respect to time is plotted in Fig. 3d. The maximum capacitance of *C*_*2*_ for successful spiking at a given capacitance of *C*_*1*_ tends to increase monotonically with that of *C*_*1*_. A higher capacitance of *C*_*1*_ allows a longer discharging time of *C*_*1*_; the discharging arises from the on-switching of switch *S*_*1*_ and continues as far as *|V*_*1*_*+V*_*d*_*|* >*V*_*off*_, i.e., off-switching of switch *S*_*1*_. A higher capacitance of *C*_*2*_ allows the charging time of *C*_*2*_ to be longer; the charging arises from an increase in *|V*_*2*_*|* (*V*_*2*_ < 0) occurring upon the prior on-switching of switch *S*_*1*_ and continues until *|V*_*2*_*–V*_*d*_*|* ≥*V*_*on*_, i.e., on-switching of switch *S*_*2*_. Thus, a higher capacitance of *C*_*1*_ enables the capacitance range of *C*_*2*_ to widen, leading to the formation of the capacitance window shown in Fig. 3a. To highlight the capacitance dependence, four *C*_*1*_ and *C*_*2*_ pairs, denoted by α, β, γ, and δ in Fig. 3a, were sampled and for each pair a “membrane potential,” i.e., *V*_*2*_, the profile with respect to time was evaluated for the given input current *I*_*in*_. The results plotted are shown in Fig. 3b and 3c. In case of δ, the charging period of capacitor *C*_*2*_ is longer than the discharging period of capacitor *C*_*1*_, and thus switch *S*_*1*_ recovers its off-state before a transition of switch *S*_*2*_ into the on-state. Therefore, no spiking is observed (see Fig. 3c). To closely look at the evolution of *V*_1_, *V*_2_, *R*_S1_ (*R* of *S*_1_), and *R*_S2_ (*R* of *S*_2_) for case β, their time-dependent behaviors are zoomed in in Fig. 3e and 3f.

The spiking dynamics is described by the membrane potential *V*_2_ and the auxiliary variable *V*_1_ as follows:


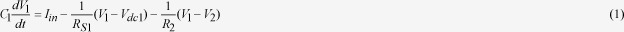


and


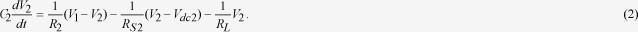


The NLIF neuron model is similar to the LIF neuron model regarding such that capacitor *C*_2_ integrates potential and fires a spike when the threshold for the on-switching of *S*_2_ is reached. However, a difference lies in the auxiliary variable *V*_1_ in Eqs. (1) and (2). Given these two variables, the spiking dynamics can be mapped onto a *V*_2_-*V*_1_ phase-plane, which is analogous to two-dimensional Hodgkin-Huxley neuron models such as FitzHugh-Nagumo model^33^,^34^. Eqs. (1) and (2) express *V*_1_- and *V*_2_-nullclines as the following linear equations:


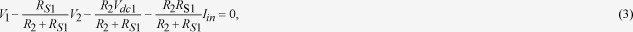


and


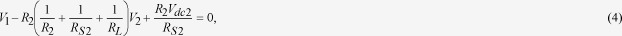


respectively. *R*_S1_ and *R*_S2_ are history- and voltage-dependent, and thus so are these nullclines. When *R*_S1_ *=* *R*_off_ and *R*_S1_ *=* *R*_on_, the *V*_1_-nullcline is given by


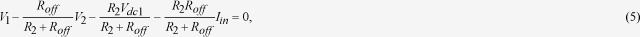


and


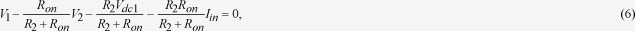


respectively. Likewise, the *V*_2_-nullcline is expressed as


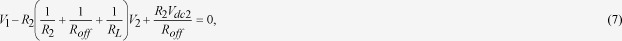


and


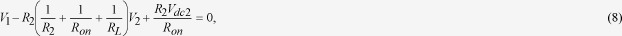


for case of *R*_S2_ *=* *R*_off_ and *R*_S2_ *=* *R*_on_, respectively.

**Figure 4:**
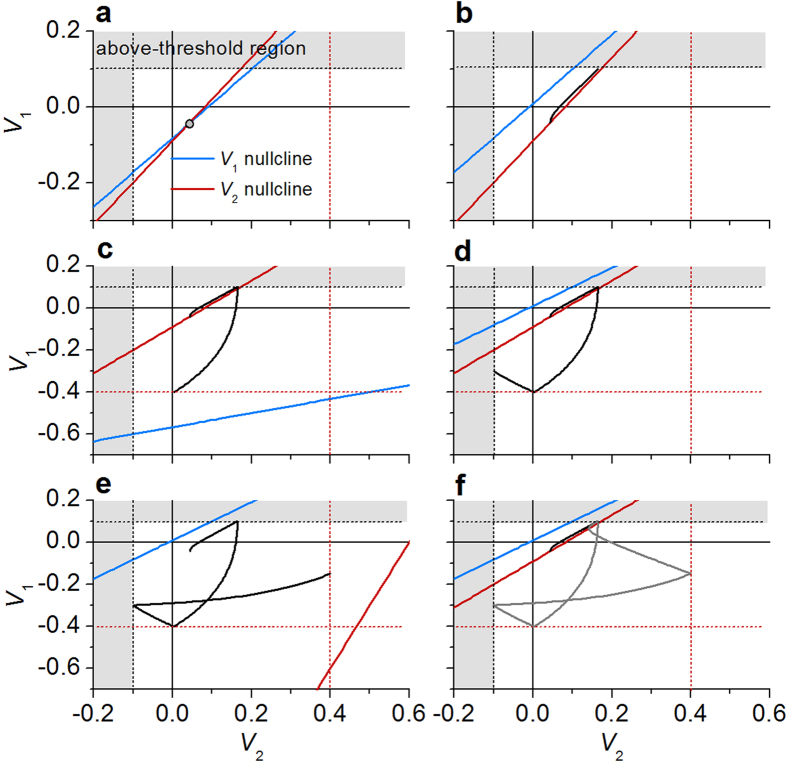
Spiking dynamics on two-dimensional phase-plane. (**a**) *V*_1_- and *V*_2_-nullcline and a stable fixed point (grey circle) when *I*_in_ *=* 0. The white area denotes the sub-threshold region. (**b**–**f**) Changes in the *V*_1_- and *V*_2_-nullcline upon threshold switching of *S*_1_ and *S*_2_ and the consequent trajectory of *V*_1_ and *V*_2_ on the phase-plane. The grey cycle in (**f**) shows the corresponding limit cycle. The black and red dashed lines mean thresholds for on- and off-switching, respectively.

When *I*_in_ *=* 0, *V*_1_ and *V*_2_ stay at a stable fixed point (*V*_2_ *=* 0.044, *V*_1_ *=* –0.042) that is indicated in Fig. 4a. The parameters used in the phase analysis are listed in Table 2, which correspond to case β shown in Fig. 3. Upon the application of a constant current, this *V*_1_-nullcline in Eq. (5) shifts upwards by 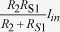 so that the fixed point moves far in the above-threshold region as shown in Fig. 4b. Consequently, the (*V*_2_, *V*_1_) trajectory moves towards the fixed point in due course as seen in Fig. 4b until the on-switching condition for *S*_1_, *V*_1_ ≥ |*V*_*on*_| + *V*_*dc*1_ *=* 0.1 V, is encountered. The other *V*_1_-nullcline in Eq. (6) emerges at this moment, leading the trajectory to a new fixed point – again outside the sub-threshold region (see Fig. 4c). On its way, the off-switching condition for *S*_1_, *V*_1_ ≤ |*V*_*off*_ | + *V*_*dc*1_ *=* −0.4 V, is met; the initial *V*_1_-nullcline (Eq. (5)) is thus recovered, changing the direction again (see Fig. 4d). The path undergoes another change when it reaches the on-switching condition for *S*_2_, *V*_2_ ≤ − |*V*_*on*_| +* V*_*dc*2_ *=* −0.1 V, as a consequence of emergence of the above-threshold *V*_2_-nullcline given by Eq. (8) (Fig. 4e). Encountering the off-switching condition for *S*_2_, *V*_2_ ≥ − |*V*_*off*_ | +* V*_*dc*2_ *=* 0.4 V, recovers the sub-threshold *V*_2_-nullcline (Eq. (7)), so that the path heads to a new fixed point as shown in Fig. 4f. The subsequent spiking dynamics follows the limit cycle that is indicated using a grey line in Fig. 4f. The spiking dynamics of the NLIF neuron differs from that of the Hodgkin-Huxley neuron mainly in the fact that a stable fixed point varies upon *V*_1_ and *V*_2_ on the phase-plane, due to the *V*_1_- and *V*_2_-nullclines conditional on *V*_1_ and *V*_2_.

Notably, the limit cycle is confined in the area (*−*|*V*_*on*_| +* V*_*dc*2_ ≤ *V*_2_ ≤ −|*V*_*off*_ | + *V*_*dc*2_ and |*V*_*off*_ | + *V*_*dc*1_ ≤ *V*_1_ ≤ |*V*_*on*_| + *V*_*dc*1_) on the phase-plane as seen in Fig. 4. That is, the spike’s height and the level of the following undershoot are determined by *V*_dc1_, *V*_dc2_, *V*_on_, and *V*_off_, so that they are important parameters in spike’s shape design.

### Neuronal selectivity of the NLIF neuron

The circuit parameters of the NLIF neuron are required to be further optimized by taking into account the neuronal selectivity of the NLIF neuron for stimulation. As an information encoder, the NLIF neuron should be able to represent “distinguishable” responses to different stimuli. Neuronal responses are typically parameterized by the spiking rate or the spike number – also known as activity – in a given time period. Regarding the neuronal selectivity, the NLIF neuron needs to vary its firing rate upon input current *I*_*in*_. In particular, the firing rate and the input current are expected to be in a one-to-one correspondence relationship. Otherwise, one can hardly estimate the stimulus by counting the number of spikes, implying difficulty in “decoding” neuronal information. This difficulty in decoding consequently reduces the amount of information conveyed by the neuron^35^. In general, a neuronal encoding process is described by *a* *=* *G*[*I*_*in*_(*s*)], where *a* and *s* denote activity and stimulus, respectively. In this study, we define neuronal “activity” denoting the number of spikes in a time period of 30 ms. The function *G* in a biological neuron is nonlinear and exhibits a threshold value for activation, i.e., firing, of the neuron. This function is often referred to as the neuronal response function in which the activity is determined by input current *I*_*in*_.

**Figure 5:**
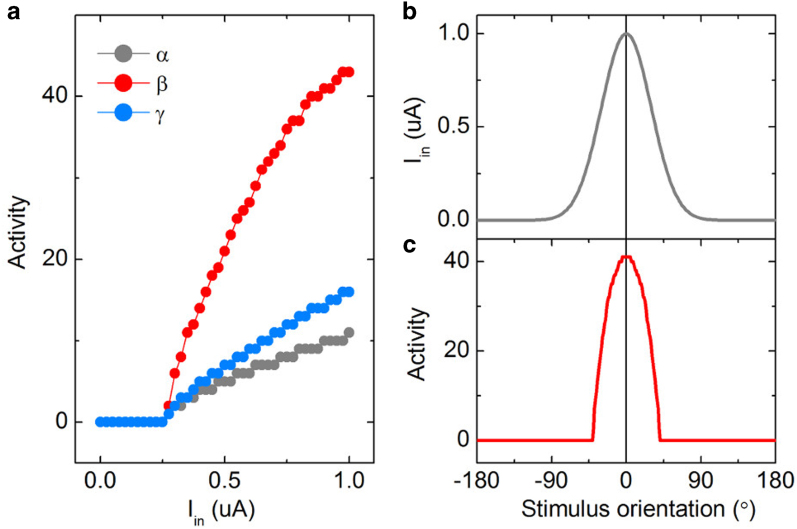
Tuning function of ideal NLIF neuron. (**a**) Neuronal response functions of the NLIF neuron with the three combinations of *C*_*1*_ and *C*_*2*_ (α, β, and γ). (**b**) A Gaussian distribution of input current *I*_*in*_, centered at a preferred orientation of 0°. (**c**) An ideal tuning curve of the NLIF neuron corresponding to the β case with a preferred orientation of 0°.

For cases of aforementioned α, β, and γ, the neuronal response functions were simulated in the input current *I*_*in*_ range (0–1.0 μA) and they are plotted in Fig. 5a. The neuronal response functions tend to increase monotonically with input current *I*_*in*_ as long as the current is larger than a threshold of approximately 0.25 μA. This threshold results from a threshold voltage for the on-switching of switches *S*_*1*_ and *S*_*2*_ (*V*_*on*_). All these functions appear to fulfill the aforementioned requirements for successful neuronal encoding. Nevertheless, a higher *da/dI*_*in*_ value is favorable considering the fact that it reduces the uncertainty in discrimination when “noisy” neuronal responses are decoded. This issue will be revisited later when dealing with the noisy behavior of the NLIF neuron. Thus, case β appears to be most favorable and further discussion in this study will be narrowed down on this particular case. The corresponding parameters are listed in Table 2.

In biological neurons, the input current *I*_*in*_ is understood to be determined by stimulus *s*, that is, *I*_*in*_ is a function of stimulus *s*. Each individual neuron has a preferred stimulus *s*_*p*_, at which *I*_*in*_ injected into a given neuron becomes the maximum. Note that this corresponds to the case of controlled neurophysiology experiments. The neuronal *in-vivo* function is, however, by and large triggered by an incident spike train(s) that provides a time-varying, rather than constant, synaptic current^1^. For simplicity, only one-dimensional stimuli are of concern in this study. For instance, stimulus *s* can be a one-dimensional visual stimulus such as the orientation angle of a light bar for the primary visual cortex^36^,^37^ and a wind direction for the cricket cercal system^38^. For convenience, the orientation of a light bar is considered as a one-dimensional stimulus in this study. It is assumed that the input current is described by a Gaussian function whose maximum is placed at preferred stimulus *s*_*p*_ as follows: 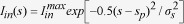, where *I*_*in*_^*max*^ and *σ*_*s*_ are the maximum *I*_*in*_ and the standard deviation, respectively. Fig. 5b shows the assumed *I*_*in*_ distribution with respect to stimulus, where *I*_*in*_^*max*^, *s*_*p*_, and *σ*_*s*_ are 1 μA, 0°, and 30°, respectively. Entering this *I*_*in*_(*s*) function into the neuronal response function *G*[*I*_*in*_] for β eventually gives the tuning curve shown in Fig. 5c. This bell-shaped tuning curve appears consistent with that obtained for typical biological neurons. The response of the NLIF neuron shows its maximum at an orientation of 0°, corresponding to its preferred orientation, and tails around the preferred orientation. That is, stimuli within an orientation range of approximately −40° to 40° are able to activate the NLIF neuron although they are not exactly coincident with the preferred orientation. As a matter of fact, this imperfect-looking tuning curve enables a population of neurons with the limited number of preferred orientations to encode continuous, i.e., analog, information^31^. If neurons represented delta-function-like tuning curves, then an infinite number of such neurons would be required for encoding analog orientation information.

### Noisy NLIF neuron

**Figure 6:**
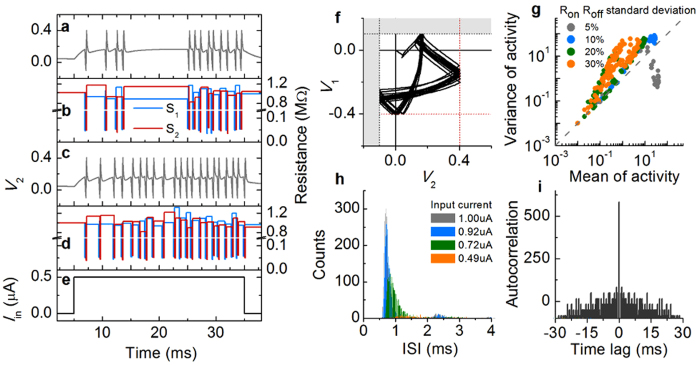
Poisson-like noise of NLIF neuron. (**a**) Noisy response of the NLIF neuron with 10 percent resistance deviation and (**b**) the corresponding fluctuation of resistance of TSs in time. (**c**) Another set of noisy response and (**d**) resistance fluctuation under the same condition. The input current for both cases is plotted in (**e**). (**f**) Spiking dynamics in (**a**), mapped onto the phase-plane for the dynamics. (**g**) Variance of activity with respect to mean activity for the four different resistance deviations (5, 10, 20, and 30 percent). (**h**) ISI distribution for the case of 10 percent of resistance deviation at four different *I*_in_ values (1.00, 0.92, 0.74, and 0.49 μA). (**i**) Autocorrelation of spikes in a train at 0.5 μA input current and 10 percent of resistance deviation.

The tuning curve shown in Fig. 5c is of a perfectly working NLIF neuron. The orientation information encoded in the neuron can be decoded without uncertainty. Now, an arising question is “how large is the impact of imperfect behavior of the NLIF neuron on neuronal encoding and decoding?” Imperfect behavior is most likely caused by the variability of switching parameters of switches *S*_*1*_ and *S*_*2*_ (*R*_*on*_, *R*_*off*_, *V*_*on*_, and *V*_*off*_). For actual experimental NLIF neurons, variations in such parameters cannot be avoided. Thus, an attempt to determine the quantitative uncertainty in processing neuronal information, caused by such variations, was made by evaluating the neuronal encoding and decoding processes with varying switching parameters. Firing each spike in a spike train involves off → on → off switching of each *S*_*1*_ and *S*_*2*_ in a consecutive order. Given that the switching parameters in an experimental switch, in general, varies on each switching cycle^24^,^26^, it is rather natural to assign different switching parameters to each switch immediately after each switching event. That is, repeating a random update on the parameters lasts throughout the entire spiking period. In this regard, the switching-event-driven randomness leads to time-varying variability, and thus “noise” rather than heterogeneity^39^. Such variations change the inter-spike interval (ISI) while a constant *I*_*in*_ is applied for a given time period. Spiking behavior involving the variation is shown in Fig. 6. For this simulation, *R*_*on*_ and *R*_*off*_ of switches *S*_*1*_ and *S*_*2*_ were randomly sampled using Gaussian PDFs – centered at 50 kohm and 1 Mohm, respectively, with various deviations. After each of on- and off-switching events, a new resistance was assigned to the switches. The switching-event-driven update therefore lets *R*_*on*_ and *R*_*off*_ fluctuate in time as shown in Fig. 6b and d, implying a noise.

It should be noted that the nullclines, Eqs. (5), (6), (7), (8), are determined by *R*_on_ and *R*_off_ of *S*_1_ and *S*_2_, and thus their variation essentially alters the nullclines and the corresponding fixed point. The random update, therefore, alters the trajectory of (*V*_2_, *V*_1_) on the phase-plane. The spiking dynamics on the phase-plane and in the corresponding time-domain is shown in Fig. 6f and 6a, respectively. In this regard, the noise of the NLIF neuron is distinguished from other noise models for LIF models, e.g. diffusive noise given white noise and/or noisy synaptic current^40^, and for conductance-based model such as Hodgkin-Huxley neuron^41^,^42^.

The observed noise characteristics were quantitatively analyzed by examining a relationship between the mean and the variance of the spike number (activity) for a time period of 30 ms (see Fig. 6g). This relationship generally explains a type of the present noise. For instance, in some biological neurons, this relationship is often given by 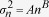 and *B*≈1, where 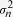 and *n* denote the variance and the mean of the activity, respectively^43^. This type of noise is referred to as the Poisson noise because the spike generation takes after the Poisson process^1^. The Poisson noise results in a straight line whose slope is unity as indicated with the dashed line in Fig. 6g. The relationship in Fig. 6g approximately features linearity – similar to the Poisson noise – albeit steeper than unity (ca. 1.3). The NLIF neuron therefore exhibits a Poisson-like, rather than perfect Poisson, noise and the variance is by and large larger than the Poisson noise at given mean activities. Interestingly, for the 5 percent deviation case, the variance is much smaller than that of the Poisson neuron at mean activities of approximately 30. This is attributed to the activity limit by the capacitors’ charging and discharging times that restrict the integration time for spiking (see Figure S1 in Supplementary Information).

The stochastic characteristics of this seemingly Poisson-like noise were further confirmed by analyzing the distribution of ISIs and the autocorrelation of the spikes in a given spike train; the results are shown in Fig. 6h and i, respectively. In Fig. 6h, the distribution is better fitted to a Gamma, rather than exponential, function regarding the effective refractory time caused by the finite recharging time of mainly capacitor *C*_1_^1^. The evaluated autocorrelation data in Fig. 6i represent typical delta-function-like distribution, suggesting no correlation between the spikes. These noise analyses, therefore, identify Poisson-like noise characteristics of the observed noise.

**Figure 7:**
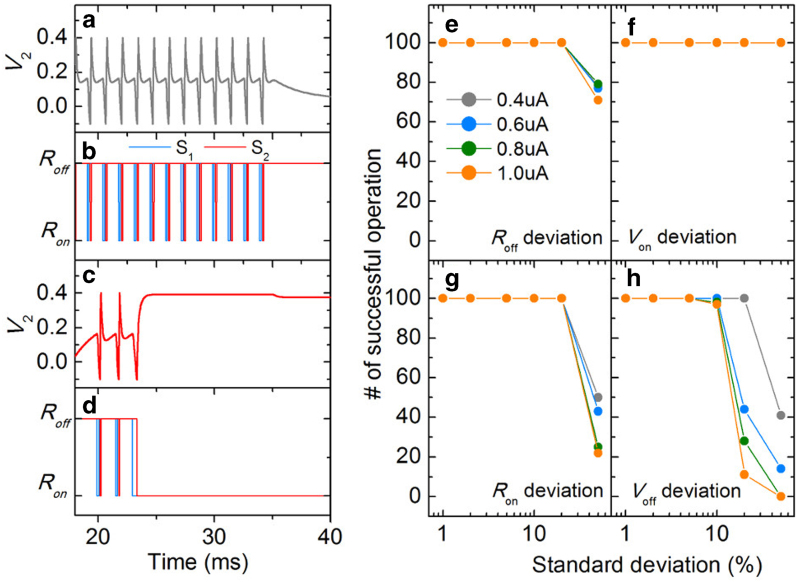
Failure of spiking. (**a**) Successful spiking example and (**b**) the corresponding variation of resistance of TSs. (**c**) Evolution of membrane potential in time in case of failure and (**d**) the corresponding change of resistance of TSs. This failure arises from switch *S*_2_ being stuck to its on-state. The number of successful spiking events on 100 trials was evaluated at given standard deviation of each switching parameter while the other parameters are fixed: (**e**) *R*_off_, (**f**) *V*_on_, (**g**) *R*_on_, and (**h**) *V*_off_. The evaluation was done at four different input currents (0.4, 0.6, 0.8, and 1.0 μA). The circuit parameters in use, encompassing the mean values of *R*_off_, *V*_on_, *R*_on_, and *V*_off_, are listed in Table 2.

To achieve successful spiking, the standard deviations of the switching parameters should be confined within particular ranges. Mostly, failure of spiking takes place when switch *S*_*2*_ becomes stuck to its on-state and high membrane potential (*V*_*2*_) is maintained. Typical examples of successful spiking and failure cases are seen in Fig. 7a and c, respectively. Insomuch as switch *S*_*2*_ keeps its on-state in case of failure, the membrane potential remains high, so that no further switching of switch *S*_*1*_ occurs. The current pulse duration was set to 30 ms. The number of successful spiking events was evaluated with separately varying the standard deviation of each switching parameter to determine the tolerance limit of each parameter (see Fig. 7e-h). We also varied input current *I*_*in*_ (0.4, 0.6, 0.8, and 1.0 μA) in order to identify its effect on success in spiking. It turned out that the tolerance limit for *R*_*on*_ and *R*_*off*_ reaches up to approximately 30 percent, whereas the limit for *V*_off_ is less than 20 percent as shown in Fig. 7e–h. It should be noted that the current pulse duration affects the probability of occurrence of an “*R*_on_-stuck” event. This happens because the longer the pulse lasts, the more spikes are likely to be emitted and the more likely that after one of these spikes switching parameters are set to a configuration that does not support further firing.

**Figure 8:**
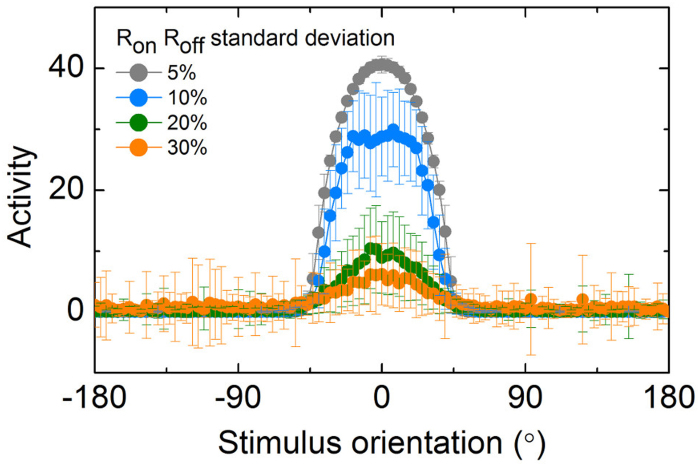
Variability effect on tuning function. Poisson-like-noise-including tuning curves of the NLIF neuron allowing 5, 10, 20, and 30 percent of resistance deviation.

The switching parameters were chosen in a random manner by employing a Gaussian probability density function (PDF) with particular standard deviations. The means of these distributions were placed at the values used in the calculation of the perfect tuning curve in Fig. 5c (*R*_*on*_: 50 kohm, *R*_*off*_: 1 Mohm, *V*_*on*_: 1 V, and *V*_*off*_: 0.5 V, the other parameters are shown in Table 2). The encoding process of a noisy individual NLIF neuron was evaluated by calculating its tuning curve based on statistics. *R*_*on*_ and *R*_*off*_ simultaneously varied at different standard deviations and the mean activity at each orientation was obtained out of 100 trials. Given the very limited tolerance for *V*_*off*_ variation as shown in Fig. 7h, no variation in *V*_*off*_ was taken into account. Variation in *V*_on_ was also ruled out in light of its negligible effect on successful operation probability (see Fig. 7f). The calculated tuning curves for four different deviations (5, 10, 20, and 30 percent) are shown in Fig. 8. The maximum activity tends to decrease with increasing standard deviation. This rapid decrease in activity results from the fact that higher deviation renders switching parameters more likely to settle into a configuration that does not support further spiking. Unexpected spiking also takes place, in particular, at orientations outside the active range (−40° - 40°). Unlike the ideal tuning curve in Fig. 5c, a one-to-one correspondence relationship between orientation and activity is no longer satisfied. Therefore, a significant difficulty in decoding the neuronal information arises, consequently reducing the amount of information conveyed by the noisy neuron^35^.

### Representation of a population of NLIF neurons

The noise in the individual NLIF neuron seems to be an obstacle to appropriate neuronal information processing because of the difficulty in decoding caused by the noise. Fortunately, neuronal information processing in the brain does not strongly rely on individual neurons; instead, the task is in general performed by a population of individual neurons^2^,^31^,^35^. Nevertheless, the neuronal noise can still contaminate the population response. Some types of correlations between neurons in a population are known to reduce errors to some extent^35^, but this does not seem to be the general case. A possible answer to the question “how do brains as groups of unreliable (noisy) neurons work reliably?” is that populations of neurons may encode and decode “probability distributions” rather than particular values^44^,^45^,^46^. In other words, encoding and decoding are viewed as processes retuning probability distributions over all possible values: response and stimulus distributions for encoding and decoding, respectively. Especially, decoding is most likely based on a statistical inference process, in particular, Bayesian inference^32^,^45^,^47^,^48^. In fact, some psychophysical evidence for Bayesian inference have been found in, for instance, contrast-depending velocity perception^45^,^49^. Given the role of the NLIF neuron in either hardware-based or *in silico* systems, it is then an important task to examine the NLIF neuron as a Bayesian decoder, quantitatively evaluating probability distributions over the orientation at given degrees of variability of the switching parameters.

According to the Bayes’ rule, a posterior PDF *P*[*s*|*r*] is given by the product of the likelihood function *P*[*r*|*s*] and the prior PDF *P*[*s*]:


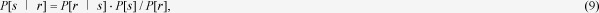


where *s* and *r* denote stimulus and response, respectively. The notation *P*[*B*|*A*] means the conditional probability of event *B*, given event *A*; the likelihood function *P*[*r*|*s*] denotes the probability of observing response *r*, given stimulus *s*. This function describes the variability of the response to a particular stimulus. Likewise, the posterior PDF *P*[*s*|*r*] means the probability of stimulus *s*, given observation of response *r*. Insomuch as no condition is imposed on the prior and the response PDF, *P*[*s*] and *P*[*r*] are constant in ranges of stimulus *s* and response *r*, respectively. Thus, the posterior can be evaluated if the likelihood is known; the maximum of the posterior PDF corresponds to the most probable stimulus *s* estimated from the response observations. That is, the observed neuronal responses can be decoded in terms of probability. Note that *P*[*s*|*r*]/*P*[*r*] denotes a normalized likelihood PDF.

Unfortunately, the NLIF neuron representing a Poisson-like noise does not allow an analytical description of its likelihood function unlike Poisson neurons^1^. The only way to obtain the likelihood is collecting the responses of a population of NLIF neurons statistically, given various stimuli acting on it. Statistics were made on 20 NLIF neurons of 20 different preferred stimuli *s*_*p*_ that are homogeneously distributed in the orientation range −180° to 180°. Now, the response *r* is a vector quantity 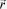 of 20 components: 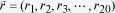. The tuning curves of these 20 neurons on the assumption of no noise are shown in Figure S2 in Supplementary Information. No correlations between neighboring neurons are assumed and the firing event on each neuron is regarded to be independent, allowing the following simple calculation:


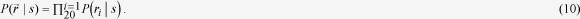


**Figure 9:**
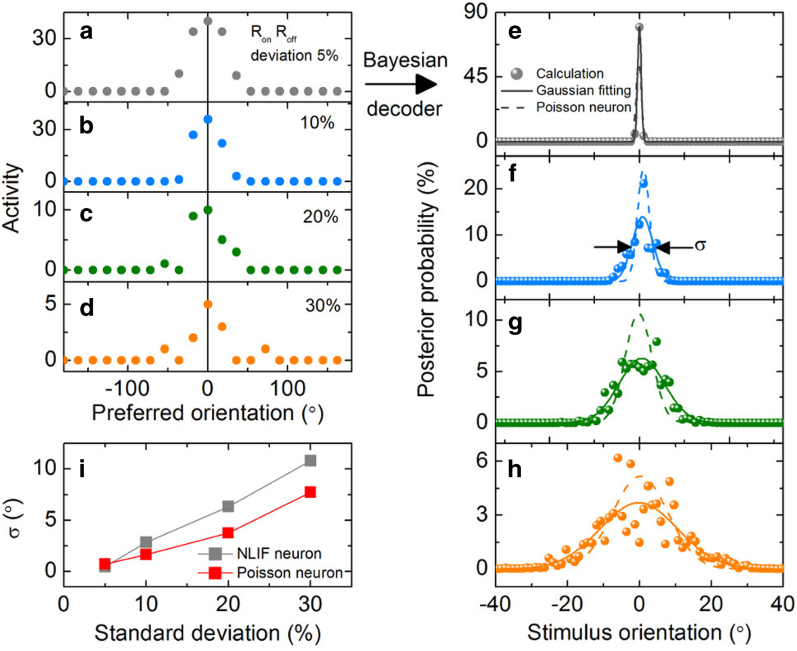
Bayesian decoding of population representation of NLIF neurons. Snapshotted activity patterns of a population including 20 NLIF neurons for (**a**) 5, (**b**) 10, (**c**) 20, (**d**) 30 percent resistance deviation cases at a stimulus of 0°. The results of the Bayesian decoding, i.e., posterior PDFs, for the patterns are shown in (**e**), (**f**), (**g**), and (**h**), respectively. The acquired posterior PDFs are compared with those of a population of 20 Poisson neurons (dashed lines). The standard deviations σ of the posterior PDFs of the Poisson-like NLIF neurons for the different resistance deviations are shown in (**i**) in comparison with those of the Poisson neurons.

The likelihood function was acquired by repeating spike number evaluation over 1000 times at each stimulus *s* and 300 stimuli were sampled between −180° and 180°. Given Eqs. (9) and (10) and the constant PDFs *P*[*s*] and *P*[*r*], the posterior PDF satisfies the condition 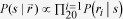. Thus, we can evaluate the probability of population representation of a particular pattern 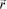 when subject to a given stimulus *s*. We snapshotted 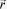 patterns of the population of NLIF neurons with resistance deviations of 5, 10, 20, and 30 percent at a stimulus of 0°, and the patterns are plotted in Fig. 9a, b, c, and d, respectively. The different preferred orientations of the population let a few neurons preferring stimuli in the vicinity of 0° be activated despite the noise complicating the patterns.

Finally, the aforementioned Bayesian decoding was done for the patterns, leading the posterior PDFs shown in Fig. 9e, f, g, and h, respectively. The posterior PDFs are more or less noisy showing data scattering; the larger the deviation, the larger the data scattering. This data scattering is also a matter of population size, i.e., the more neurons in the population, the less decoding error. Given a large increase in calculation time with increasing the number of neurons in the population, we placed 20 neurons in the population; however, the Bayesian decoding of larger population sizes definitely enables correct estimation. The calculated posterior PDFs were fitted using Gaussian PDFs so as to evaluate the center and standard deviation of each posterior PDF. As can be seen in Fig. 9, the center of each PDF is found to be placed around 0°. This revealed that this Bayesian decoder most likely give a correct answer and a correct inference will be made if made by means of the Bayes’ rule despite the present Poisson-like noise. Nevertheless, note that statistics cannot be free from error in any cases so that the Bayesian decoding can give a wrong answer at times. Besides, it turns out that the standard deviation in the decoding becomes larger as increasing the variability of R_on_ and R_off_ of the TSs (see Fig. 9i).

The Bayesian decoding results were compared with the case of populations of Poisson neurons. The likelihood PDF as well as the posterior PDF of a population of independent Poisson neurons is given by a closed-form expression; an increase in the number of Poisson neurons in the population leads to a posterior PDF of a Gaussian form^32^. At a given stimulus, the likelihood PDF of each independent Poisson neuron was analytically calculated with similar activity as that of the NLIF neuron at the same stimulus. As a result, the posterior PDF of a population of Poisson neurons with the four resistance deviations could be obtained from the population response patterns 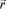 shown in Fig. 9a, b, c, and d; the calculated PDFs are plotted using dashed lines in Fig. 9e, f, g, and h, respectively. In fact, the 20 Poisson neurons already provide a Gaussian PDF as shown in the figures. In comparison with the population of Poisson neurons, it is noticed that the Poisson-like NLIF neurons represent smaller maxima and larger deviations than the Poisson neurons under the same condition except the case of 5 percent deviation of TSs’ resistance (see Fig. 9a). A difference in the standard deviation of the posterior PDF between the NLIF neurons and the Poisson neurons is observed in Fig. 9i. The larger uncertainty deviation of the Bayesian decoding for the NLIF neurons arises from the larger deviation of activity of the NLIF neuron than that of a Poisson neuron as shown in Fig. 6g. Likewise, the larger maximum of posterior PDF of the NLIF neurons in Fig. 9e than the Poisson neurons is understood in terms of the smaller variance of activity at 5 percent deviation of TSs’ resistance at the high mean activities, shown in Fig. 6g. The smaller variance is attributed to the activity limit by charging and discharging of the capacitors.

## Discussion

The NLIF neuron studied in this work can serve as a prototypical *in silico* neuron model exhibiting a Poisson-like noise. The circuitry is simple and perhaps easy enough to be implemented in large-scale ANNs. In particular, as a result of this study, it is understood that the variability of the TSs’ resistance leads to such a Poisson-like noise that the noise behavior of this prototypical *in silico* neuron needs to be under control and appropriately designed to meet the noise behavior required for ANNs built for specific purposes.

Nevertheless, when it comes to hardware realization of such NLIF neurons, there are several practical obstacles that should be overcome to realize the goal. What is of significant importance in the Poisson-like NLIF neuronal behavior is the minimum variability of *V*_*off*_ of the TS in the NLIF neuron. As discussed earlier, the tolerance limit of *V*_*off*_ is merely a few percent unlike that of the other switching parameters, i.e., *R*_*on*_, *R*_*off*_, and *V*_*on*_. Thus, “reliability” of this unreliable neuron requires meeting this stringent requirement for ensuring reliable operation. Apart from this restriction, other requirements discussed earlier may be satisfied by appropriate choices of TS materials, systems, and their design. Another important issue that potentially hinders practical use of this type of neuron is the long-term reliability of switches *S*_*1*_ and *S*_*2*_, which are subject to the relatively high dc-voltage stress (*V*_*dc1*_ and *V*_*dc2*_). The dc voltages allow active operation of the NLIF neuron, working as effective power suppliers. Regarding the limit cycle confined in the area – (−|*V*_*on*_|+*V*_*dc*2_ ≤ *V*_2_ ≤ −|*V*_*off*_ | + *V*_*dc*2_ and |*V*_*off*_ | + *V*_*dc*1_ ≤ *V*_1_ ≤ |*V*_*on*_| + *V*_*dc*1_) on the phase-plane (see Fig. 4) – dc voltages close to, but smaller than, *V*_*on*_ need to be applied to switches *S*_*1*_ and *S*_*2*_, and thus the consequent electrical stress most likely affects the switches adversely. Eventually, it most likely leads to dielectric breakdown when a dielectric layer is in use as a TS material. This issue is also directly related to a high power consumption problem. The constant application of dc voltages during the lifetime of the NLIF neuron gives rise to severe power consumption, which is definitely against one of the inherent advantages of neuromorphic systems over standard digital systems, i.e., low power consumption. Therefore, addressing these significant problems properly accelerates practical use of such NLIF neurons in hardware-based neuromorphic systems.

The most crucial conclusion drawn from this study is that the potential variability of behavior of the TS is allowed up to a certain level as long as the Bayesian decoder is able to discriminate the encoded information correctly. In addition, the uncertainty, i.e., standard deviation, of the posterior PDF shrinks when introducing a larger number of NLIF neurons in the population. In general, the statistical accuracy of a survey increases with the number of samples. Thus, the uncertainty of posterior and likelihood of individual NLIF neurons is compensated by the increase in accuracy. An increase in the number of neurons in the population, therefore, tolerates a larger variability of switching parameters of the TSs. Nevertheless, confining the variability within a tolerance range is still of significant importance, especially confining that of *V*_*on*_.

## Methods

### Neuronal response function calculation

Eqs. (1) and (2) were solved by employing the Crank-Nicolson method. The equations are described by the following discrete forms:


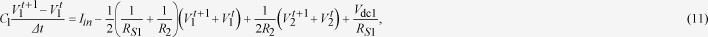


and


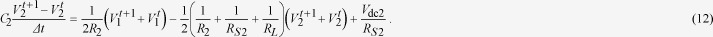


The superscripts of *V*_1_ and *V*_2_ denote the *i*th node in the time domain. Eqs. (11) and (12) were numerically solved and *R*_S1_ and *R*_S2_ were updated when the evaluated potential reaches the threshold switching conditions.

## Additional Information

**How to cite this article**: Lim, H. *et al*. Reliability of neuronal information conveyed by unreliable neuristor-based leaky integrate-and-fire neurons: a model study. *Sci. Rep*. **5**, 9776; doi: 10.1038/srep09776 (2015).

## Supplementary Material

Supplementary Information

## Figures and Tables

**Table 1:** Parameters used in the optimization of the operational window.

Symbol	Note	Value	Reference
*R*_off_*	Parameter	1 MΩ	24,27
*R*_on_	Variable	-	
*V*_off_	Variable	-	
*V*_on_	Parameter	1.0 V	26,29

^*^This value is dependent on the geometry of the TS, e.g. TS area, the thickness of the switching layer.

**Table 2:** Parameters used in the neuronal response function and tuning function simulation.

*R*_2_ [Ω]	*R*_L_ [Ω]	*R*_off_ [Ω]	*R*_on_ [Ω]	V_on_ [V]	V_off_ [V]
100 k	1 G	1 M	50 k	1.0	0.5
*V*_d_ [V]	*C*_1_ [nF]	*C*_2_ [nF]	*I*_in_^max^ [μA]	*σ*_s_ [degree]	
0.9	3	2	1	30	

## References

[b1] Dayan P. & Abbott L. F. Theoretical Neuroscience The MIT Press: London, 2001).

[b2] Eliasmith C. & Anderson C. H. Neural Engineering: Computation, Representation, and Dynamics in Neurobiological Systems MIT Press: London, 2003).

[b3] Gerstner W. & Kistler W. M. Spiking Neuron Models: Single Neurons, Populations, Plasticity Cambridge University Press: Cambridge, 2002).

[b4] Seung H. S. Connectome: How the Brain’s Wiring Make Us Who We Are Houghton Miffin Harcout: Boston, 2012).

[b5] Mead C. Neuromorphic electronic systems. Proc. IEEE 78, 1629–1636 (1990).

[b6] Eliasmith C. . A Large-Scale Model of the Functioning Brain. Science 338, 1202–1205 (2012). 23197532 10.1126/science.1225266

[b7] Merolla P. . A digital neurosynaptic core using embedded crossbar memory with 45pJ per spike in 45nm. Custom Integrated Circuits Conference (CICC), 2011 IEEE. DOI: 10.1109/CICC.2011.6055294 (2011).

[b8] Hinton G., Dayan P., Frey B. & Neal R. The “wake-sleep” algorithm for unsupervised neural networks. Science 268, 1158–1161 (1995). 7761831 10.1126/science.7761831

[b9] LeCun Y., Kavukcuoglu K. & Farabet C. Convolutional networks and applications in vision. Proc. 2010 IEEE International Symposium on Circuits and Systems (ISCAS). DOI: 10.1109/ISCAS.2010.5537907 (2010)

[b10] Markram H. The Blue Brain Project. Nat. Rev. Neurosci. 7, 153–160 (2006). 16429124 10.1038/nrn1848

[b11] Danchin A., Médigue C., Gascuel O., Soldano H. & Hénaut A. From data banks to data bases. Res. Microbiol. 142, 913–916 (1991). 1784830 10.1016/0923-2508(91)90073-j

[b12] Jeong D. S., Kim I., Ziegler M. & Kohlstedt H. Towards artificial neurons and synapses: a materials point of view. RSC Adv. 3, 3169–3183 (2013).

[b13] Hodgkin A. L. & Huxley A. F. A quantitative description of membrane current and its application to conduction and excitation in nerve. J. Physiol. 117, 500–544 (1952). 12991237 10.1113/jphysiol.1952.sp004764 PMC1392413

[b14] Izhikevich E. M. Simple model of spiking neurons. IEEE Trans. Neural Netw. 14, 1569–1572 (2003). 18244602 10.1109/TNN.2003.820440

[b15] Izhikevich E. M. Which model to use for cortical spiking neurons? IEEE Trans. Neural Netw. 15, 1063–1070 (2004). 15484883 10.1109/TNN.2004.832719

[b16] Indiveri G., Chicca E. & Douglas R. A VLSI array of low-power spiking neurons and bistable synapses with spike-timing dependent plasticity. IEEE Trans. Neural Netw. 17, 211–221 (2006). 16526488 10.1109/TNN.2005.860850

[b17] Indiveri G. . Neuromorphic silicon neuron circuits. Front. Neurosci. 5, 1–23 (2011). 21747754 10.3389/fnins.2011.00073 PMC3130465

[b18] Pickett M. D., Medeiros-Ribeiro G. & Williams R. S. A scalable neuristor built with Mott memristors. Nature Mater. 12, 114–117 (2012). 23241533 10.1038/nmat3510

[b19] Crane H. D. Neuristor-A novel device and system concept. Proc. IRE 50, 2048–2060 (1962).

[b20] Mott N. F. The Basis of the Electron Theory of Metals, with Special Reference to the Transition Metals. Proc. Phys. Soc. Sect. A 62, 416–422 (1949).

[b21] Jeong D. S. . Emerging memories: resistive switching mechanisms and current status. Rep. Prog. Phys. 75, 076502 (2012). 22790779 10.1088/0034-4885/75/7/076502

[b22] Cario L., Vaju C., Corraze B., Guiot V. & Janod E. Electric-field-induced resistive switching in a family of Mott insulators: towards a new class of RRAM memories. Adv. Mater. 22, 5193–5197 (2010). 20957700 10.1002/adma.201002521

[b23] Pickett M. D. & Williams R. S. Sub-100 fJ and sub-nanosecond thermally driven threshold switching in niobium oxide crosspoint nanodevices. Nanotechnol. 23, 215202 (2012). 10.1088/0957-4484/23/21/215202 22551985

[b24] Jeong D. S. . Threshold resistive and capacitive switching behavior in binary amorphous GeSe. J. Appl. Phys. 111, 102807 (2012).

[b25] Ovshinsky S. R. Reversible electrical switching phenomena in disordered structures. Phys. Rev. Lett. 21, 1450–1453 (1968).

[b26] Lee M.-J. . A plasma-treated chalcogenide switch device for stackable scalable 3D nanoscale memory. Nat. Commun. 4, 2629 (2013). 24129660 10.1038/ncomms3629

[b27] Ahn H.-W. . A Study on the Scalability of a Selector Device Using Threshold Switching in Pt/GeSe/Pt. ECS Solid State Letters 2, N31–N33 (2013).

[b28] Han J.-W. & Choi Y.-K. Bistable resistor (biristor) - gateless silicon nanowire memory. 2010 Symposium on VLSI Technology. DOI: 10.1109/VLSIT.2010.5556215 (2010).

[b29] Liu X. . Diode-less bilayer oxide (WOx –NbOx) device for cross-point resistive memory applications. Nanotechnol. 22, 475702 (2011). 10.1088/0957-4484/22/47/475702 22056387

[b30] Deneve S., Latham P. E. & Pouget A. Reading population codes: a neural implementation of ideal observers. Nat. Neurosci. 2, 740–745 (1999). 10412064 10.1038/11205

[b31] Pouget A., Dayan P. & Zemel R. Information processing with population codes. Nat. Rev. Neurosci. 1, 125–132 (2000). 11252775 10.1038/35039062

[b32] Ma W. J., Beck J. M., Latham P. E. & Pouget A. Bayesian inference with probabilistic population codes. Nat. Neurosci. 9, 1432–1438 (2006). 17057707 10.1038/nn1790

[b33] FitzHugh R. Impulses and Physiological States in Theoretical Models of Nerve Membrane. Biophys. J. 1, 445–466 19431309 10.1016/s0006-3495(61)86902-6 PMC1366333

[b34] Nagumo J., Arimoto S. & Yoshizawa S. An Active Pulse Transmission Line Simulating Nerve Axon. Proc. IRE 50, 2061–2070 (1962).

[b35] Averbeck B. B., Latham P. E. & Pouget A. Neural correlations, population coding and computation. Nat. Rev. Neurosci. 7, 358–366 (2006). 16760916 10.1038/nrn1888

[b36] Hubel D. H. & Wiesel T. N. Receptive fields and functional architecture of monkey striate cortex. J. Physiol. 195, 215–243 (1968). 4966457 10.1113/jphysiol.1968.sp008455 PMC1557912

[b37] Henry G. H., Dreher B. & Bishop P. O. Orientation specificity of cells in cat striate cortex. J. Neurophysiol. 37, 1394–1409 (1974). 4436709 10.1152/jn.1974.37.6.1394

[b38] Theunissen F. E. & Miller J. P. Representation of sensory information in the cricket cercal sensory system. II. Information theoretic calculation of system accuracy and optimal tuning-curve widths of four primary interneurons. J. Neurophysiol. 66, 1690–1703 (1991). 1765802 10.1152/jn.1991.66.5.1690

[b39] Hamilton T. J., Afshar S., van Schaik A. & Tapson J. Stochastic Electronics: A Neuro-Inspired Design Paradigm for Integrated Circuits. Proc. IEEE 102, 843–859 (2014).

[b40] Brunel N., Chance F., Fourcaud N. & Abbott L. Effects of Synaptic Noise and Filtering on the Frequency Response of Spiking Neurons. Phys. Rev. Lett. 86, 2186–2189 (2001). 11289886 10.1103/PhysRevLett.86.2186

[b41] Gutkin B., Jost J. & Tuckwell H. Inhibition of rhythmic neural spiking by noise: the occurrence of a minimum in activity with increasing noise. Naturwissenschaften 96, 1091–1097 (2009). 19513592 10.1007/s00114-009-0570-5 PMC2727367

[b42] Tuckwell H. C. & Jost J. Weak Noise in Neurons May Powerfully Inhibit the Generation of Repetitive Spiking but Not Its Propagation. PLoS Comput Biol 6, e1000794 (2010). 20523741 10.1371/journal.pcbi.1000794 PMC2877724

[b43] Calvin W. H. & Stevens C. F. Synaptic noise as a source of variability in the interval between action potentials. Science 155, 842–844 (1967). 6018196 10.1126/science.155.3764.842

[b44] Anderson C. & Van Essen D. [Neurobiological computational systems] Computational Intelligence Imitating Life [213–222] (IEEE Press: New York, 1994).

[b45] Pouget A., Dayan P. & Zemel R. S. Inference and computation with population codes. Annu. Rev. Neurosci. 26, 381–410 (2003). 12704222 10.1146/annurev.neuro.26.041002.131112

[b46] Weiss Y. & Fleet D. J. [Velocity Likelihoods in Biological and Machine Vision] Statistical Theories of the Cortex [77–96] MIT Press: London, 2002).

[b47] Knill D. C. & Richards W. Perception as Bayesian Inference (Cambridge Univ. Press: Cambridge, 1996).

[b48] Knill D. C. & Pouget A. The Bayesian brain: the role of uncertainty in neural coding and computation. Trends Neurosci. 27, 712–719 (2004). 15541511 10.1016/j.tins.2004.10.007

[b49] Weiss Y., Simoncelli E. P. & Adelson E. H. Motion illusions as optimal percepts. Nat. Neurosci. 5, 598–604 (2002). 12021763 10.1038/nn0602-858

