# Supplementary material for: Reliability of neuronal information conveyed by unreliable neuristor-based leaky integrate-and-fire neurons: a model study
Source: Sci Rep. 2015 May 13;5:9776. doi: 10.1038/srep09776 (PMC4429369; doi:10.1038/srep09776)
Supplement: Supplementary Information [file srep09776-s1.pdf]

Supplementary Information for

Reliability of neuronal information conveyed by unreliable neuristor-based leaky integrate-and-fire neurons: a model study

Hyungkwang Lim,[1,2] Vladimir Kornijcuk,[1,3] Jun Yeong Seok,[1,2] Seong Keun Kim,[1] Inho Kim,[1] Cheol Seong Hwang,[2] and Doo Seok Jeong*[1]

[1]Electronic Materials Research Center, Korea Institute of Science and Technology, 136-791 Seoul, Republic of Korea

[2]Department of Materials Science and Engineering and Inter-university Semiconductor Research Center, Seoul National University, 151-744 Seoul, Republic of Korea

[3]Department of Materials Science and Engineering, Seoul National University of Science and Technology, 139-743 Seoul, Republic of Korea

*Correspondence and requests for materials should be addressed to D.S.J. (dsjeong@kist.re.kr)

SI1. Activity limit

Spike firing in due course in the NLIF neuron significantly depends on charging and discharging of capacitors C_1 and C_2 as the flicker of switch S_2 during its operation results from consecutive charging and discharging of capacitor C_2. It takes a particular time for capacitors to be charged and discharged, which is defined as an RC time constant. It is therefore predictable that the maximum number of spikes over a period of given time (here, 30 ms) is limited by the capacitive behavior. This limit is seen when it comes to high activity, for instance, the NLIF neuron with 5 percent R_{on} and R_{off} deviation (see Figure 6g). A drastic decrease in variance at high mean activities (> ca. 20) can be seen, implying that the variance is no longer determined by the deviation of the resistance. To highlight this activity limit, distributions of activity triggered by two different input currents (0.4 and 1.0 μA) are acquired for different R_{on} and R_{off} deviations as shown in Figures S1a and b. Considering the spike firing characteristics shown in Figure 6g, in general, a higher current as well as a smaller resistance deviation results in a larger mean activity. However, when the activity becomes close to the limit (ca. 45), the activity limit no longer allows higher activity, so that

the variance is largely suppressed as shown in Figure S1b. The limit effect on variance is obviously seen in the comparison between the two different input current cases shown in Figures S1c and d, in which the variance of the 1.0 μA injection case is largely reduced. For a comparison with the Poisson neuron, the Fano factor, denoting a variance to mean ratio, is evaluated for the two different current injection cases with respect to assumed resistance deviation (see Figure S1e). The Poisson neuron exhibits a Fano factor of unity as indicated using a dashed line in Figure S1e.[1] Unlike the perfect Poisson neuron, the Fano factor of the NLIF neuron varies upon the resistance deviation. In particular, at higher resistance deviations the Fano factor is larger than unity; the variance is larger than the mean activity, and thus the variance of the NLIF neuron is larger than that of the Poisson neuron at a given mean activity. This leads to a larger uncertainty in the Bayesian decoding than the Poisson neuron case as shown in Figure 9.

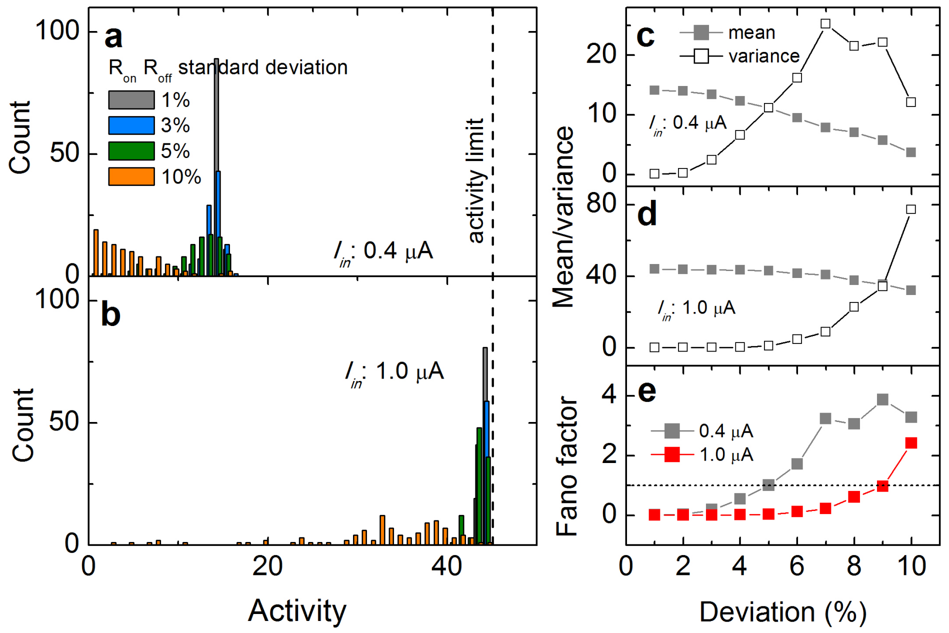

Figure S1. Histrograms of activity for cases of (a) low mean activity (I_{in}: 0.4 μA) and (b) high mean activity (I_{in}: 1.0 μA), which were acquired over 100 time trials. The dashed line in the left panel indicates the activity limit. The mean and variance of activity for different resistance deviations are plotted for cases of (c) low (I_{in}: 0.4 μA) and (d) high (I_{in}: 1.0 μA) current injection. (e) The Fano factors, i.e. variance/mean ratio, for the two different current injection cases are shown with respect to resistive deviation.

SI2. Injected current into each NLIF neuron in a population and its response

For each NLIF neuron in a population of 20 NLIF neurons, injected current with respect to orientation is plotted in Figure S2a, which represents a bell-shape curve. And the corresponding tuning curve of the NLIF neuron is shown in Figure S2b. The tuning curves were evaluated on the assumption of no deviation of R_{on} and R_{off}.

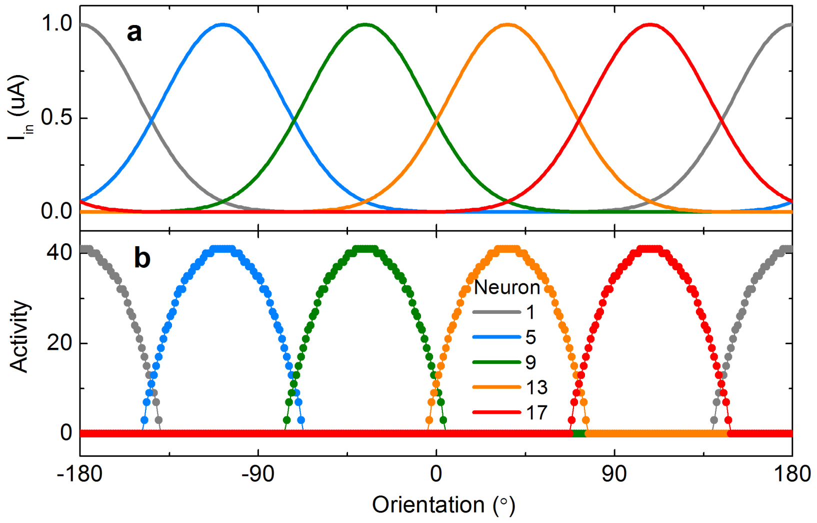

Figure S2. (a) Injected current into each NLIF neuron with an each preferred orientation in a population of 20 neurons in total. Current profiles of only 5 neurons among 20 ones in total are plotted in this figure. (b) Tuning curves of the sampled 5 NLIF neurons responding to the injected current shown in (a). Note that no resistance deviation was assumed in this calculation.

1. Dayan, P. & Abbott, L. F. *Theoretical Neuroscience*. (The MIT Press, 2001).
